# Characterization of diverse homoserine lactone synthases in *Escherichia coli*

**DOI:** 10.1371/journal.pone.0202294

**Published:** 2018-08-23

**Authors:** René Daer, Cassandra M. Barrett, Ernesto Luna Melendez, Jiaqi Wu, Stefan J. Tekel, Jimmy Xu, Brady Dennison, Ryan Muller, Karmella A. Haynes

**Affiliations:** 1 Arizona State University, School of Biological and Health Systems Engineering, Tempe, AZ, United States of America; 2 School of Molecular Sciences, Arizona State University, Tempe, AZ, United States of America; 3 School of Computing, Informatics, and Decision Systems Engineering, Arizona State University, Tempe, AZ, United States of America; 4 School of Life Sciences, Arizona State University, Tempe, AZ, United States of America; 5 School of Engineering of Matter, Transport, and Energy, Arizona State University, Tempe, AZ, United States of America; 6 Department of Chemistry and Biochemistry, Arizona State University, Tempe, AZ, United States of America; Oswaldo Cruz Foundation, BRAZIL

## Abstract

Quorum sensing networks have been identified in over one hundred bacterial species to date. A subset of these networks regulate group behaviors, such as bioluminescence, virulence, and biofilm formation, by sending and receiving small molecules called homoserine lactones (HSLs). Bioengineers have incorporated quorum sensing pathways into genetic circuits to connect logical operations. However, the development of higher-order genetic circuitry is inhibited by crosstalk, in which one quorum sensing network responds to HSLs produced by a different network. Here, we report the construction and characterization of a library of ten synthases including some that are expected to produce HSLs that are incompatible with the Lux pathway, and therefore show no crosstalk. We demonstrated their function in a common lab chassis, *Escherichia coli* BL21, and in two contexts, liquid and solid agar cultures, using decoupled Sender and Receiver pathways. We observed weak or strong stimulation of a Lux receiver by longer-chain or shorter-chain HSL-generating Senders, respectively. We also considered the under-investigated risk of unintentional release of incompletely deactivated HSLs in biological waste. We found that HSL-enriched media treated with bleach were still bioactive, while autoclaving deactivates LuxR induction. This work represents the most extensive comparison of quorum signaling synthases to date and greatly expands the bacterial signaling toolkit while recommending practices for disposal based on empirical, quantitative evidence.

## Introduction

**Fig 1:**
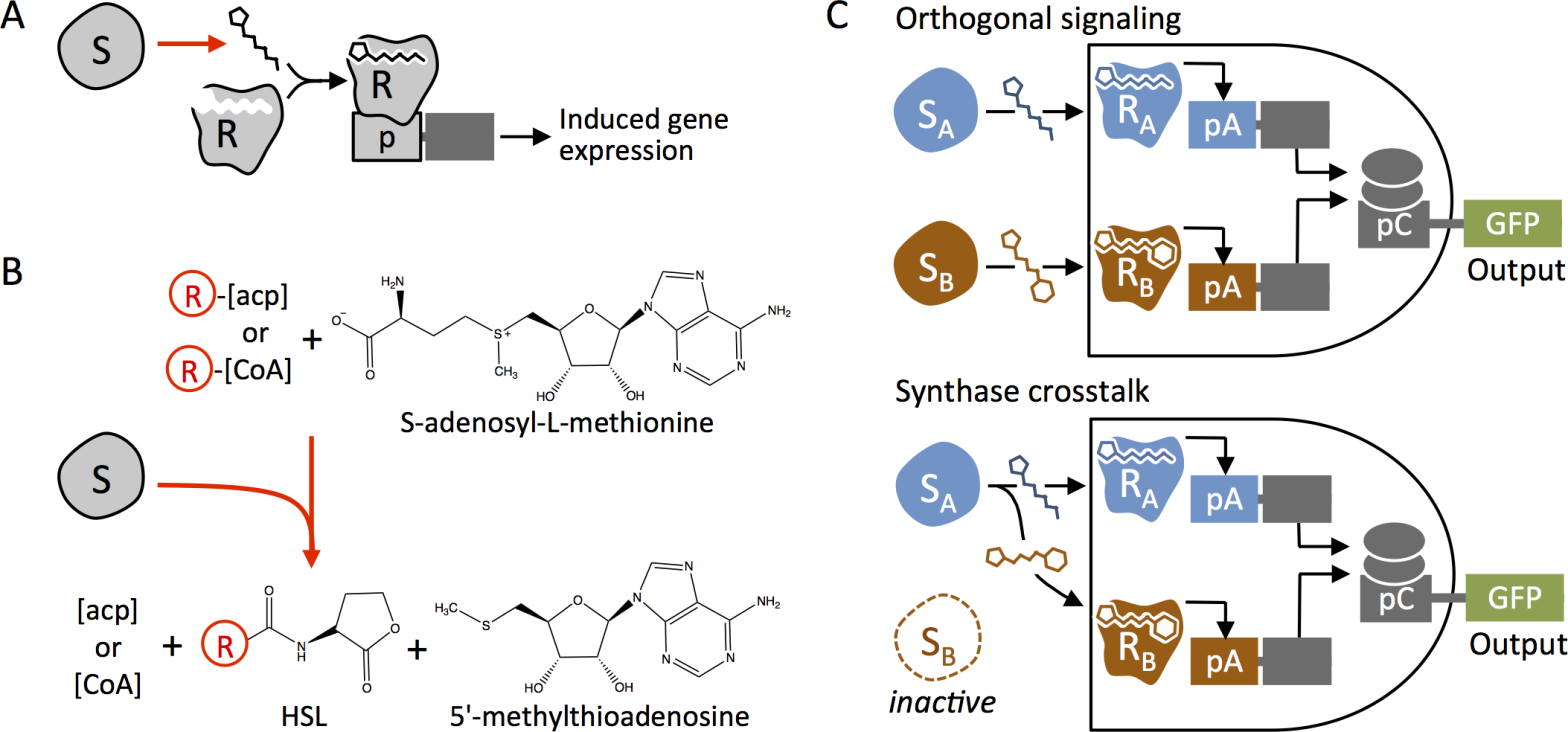
Homoserine lactone (HSL) function, production, and role in quorum sensing crosstalk. (A) Synthase protein (S) catalyzes formation of the HSL species. Regulator protein (R) complexes with the HSL ligand and binds to the promoter, which induces expression of downstream genes. (B) Synthase proteins catalyze the production of HSL molecules by facilitating lactone ring formation from S-adenosyl methionine and attaching a variable R-group tail to the lactone ring from one of two donors, acyl-carrier proteins (ACP) or coenzyme A (CoA). (C) The cartoon illustrates an example of the consequence of crosstalk. Two pathways, S_A_ > R_A_ and S_B_ > R_B_ are designed to operate in parallel as a Boolean AND gate sensor. When each synthase generates a single predominant HSL, the output indicates the activity of both S_A_ and S_B_ as intended. However, if S_A_ produces high levels of primary and secondary products that stimulate both R_A_ and R_B_, a fault occurs where output is produced although the AND condition is not met.

Quorum sensing networks enable bacteria to monitor and respond to changes in their population density by coupling gene regulation with diffusible chemical signals from neighboring bacteria [1]. These signaling networks control group behaviors such as virulence, biofilm formation, and motility [2,3]. One class of these chemical signals, known as homoserine lactones (HSLs), is produced by a family of synthase enzymes called LuxI-like proteins [4–6]. HSLs have traditionally been referred to as N-acyl homoserine lactones (AHLs). However synthases also produce non-acyl chain homoserine lactones, so in this report we use the general term HSL. Accumulation of HSLs results in activation of a DNA binding, regulator protein, or LuxR-like protein, that controls expression of genes (Fig 1A) involved in microbial population behavior. Homologous HSL networks have been identified in over one hundred species of bacteria and the discovery of more networks is ongoing [7,8]. Each network includes an HSL synthase protein that catalyzes the synthesis of specific HSL signaling molecules (Fig 1B), a regulator that is allosterically regulated by the HSL ligand, and a promoter that typically contains a palindromic sequence that is bound by the HSL-regulator complex [9,10].

Scientists have taken advantage of the simplicity of these systems to incorporate signal processing pathways into gene circuits as genetic wires to convert an output from one computation into an input of another [11]. In an engineered system, the synthase protein can be considered a “Sender” module which produces the input for a “Receiver module” comprised of the regulator and the inducible promoter upstream of an output, such as GFP [12]. Engineered quorum sensing networks are used for a variety of applications including metabolic engineering, computational circuits, and medicine. Engineered quorum sensing systems that incorporate HSL senders, rather than exogenously added synthetic HSLs, allow researchers to increase the computational complexity of a circuit. If a system employs multiple, non-overlapping quorum sensing networks, simultaneous parallel computation can occur within a single cell or linked across populations in co-culture or in solid agar [13–18]. Several examples of successful implementation of multiple networks in one circuit have been reported, however, these examples are limited to two networks in parallel and took significant efforts to optimize [11].

Promiscuity and crosstalk can occur at different steps in quorum sensing pathways, including non-specific interactions between the HSL-ligands and regulators, as well as interactions of Regulator-HSL complexes with promoters [19–22]. To mitigate this challenge, much effort has been invested in identifying or engineering orthogonal quorum sensing networks [23]. In this study we address crosstalk due to context-specific synthase activity, where a single synthase can generate and unexpected profile of HSL molecules (Fig 1C).

Natural diversity arises from variability in the HSL signal or signals employed by each network, specifically, variability in the R-groups on the lactone rings [11]. Recent efforts have led to the identification and engineering of regulators with greater specificity for chemically-distinct HSL ligands [23]. Reliable implementation of these new tools without ligand crosstalk requires complementary synthases to generate the expected HSL and minimal or no secondary products. While many synthases are known to produce a single HSL product in their native species, it cannot be assumed that they will behave similarly when expressed exogenously. Transgenic HSL synthases use endogenous acyl-carrier protein (ACP) or coenzyme A (CoA) donors as substrates to catalyze HSL formation (Fig 1B). This suggests that even with proper protein expression and folding, production of the expected HSLs depends on the availability of the appropriate ACP or CoA donor, which varies across bacterial species [24–26]. Indeed, previous work has demonstrated that some HSL synthases produce multiple HSLs [27] and that the HSL production profile can differ depending on the chassis the synthase is expressed in [28]. It is important to test sender and receiver devices in the chassis and context relevant to the intended application.

Crosstalk between species of quorum sensing bacteria is prevalent in nature as well. Environmental bacteria can respond to HSLs produced by other species for both cooperative and competitive gains [29–31]. The generation and disposal of large quantities of HSLs could cause misregulation of quorum sensing within microbial niches. Quorum sensing networks are ubiquitous and crucial to many natural systems; they coordinate virulence in human pathogens [29,32], maintain the exchange of nutrients between nitrogen-fixing Rhizobia and legumes [33], and regulate signaling between photosynthetic symbionts and their reef coral hosts [34]. It is therefore necessary to critically evaluate conventional methods for bacterial culture disposal to determine whether they are sufficient for deactivating HSLs in order to mitigate potential health risks and environmental impacts.

## Results

### Construction and expression of a HSL synthase library

**Fig 2:**
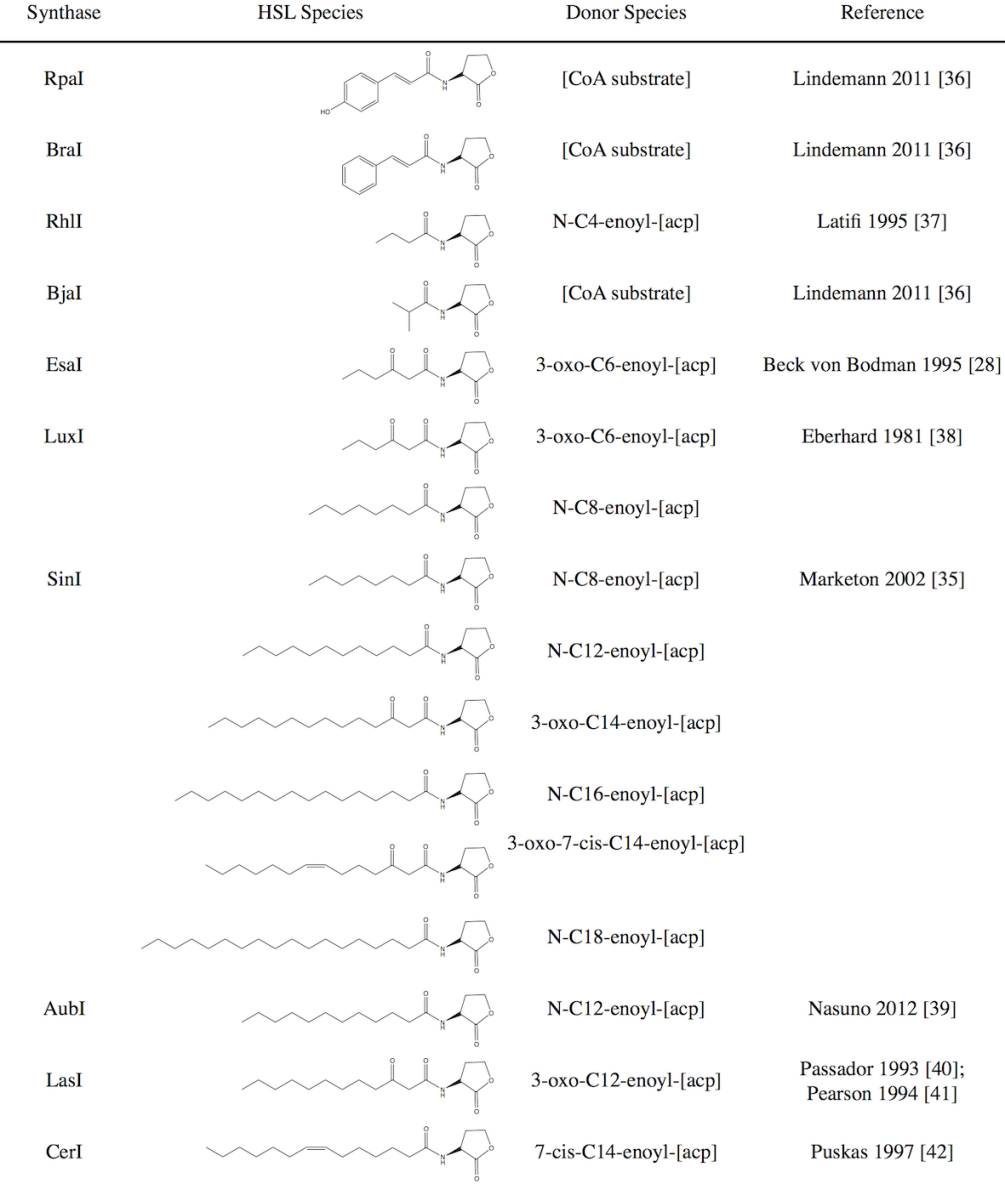
Synthases used in this study. The table lists the synthase symbol, the structures of the homoserine lactone (HSL) species produced by the native organism, and the acyl-carrier protein (ACP) or coenzyme A (CoA) donor species catalyzed by the synthase. [28,35–42].

Minimizing crosstalk between quorum sensing networks will enable genetic engineers to operate quorum sensing pathways in parallel, and therefore build more complex circuits. Toward this end, we selected ten HSL synthase genes that are known produce chemically-diverse signaling molecules with few secondary products in their native species. We considered the length of the acyl chain, the chain saturation, and the number of different HSLs produced by a single synthase. From our previously published list of reported synthase proteins and their HSL profiles [11], we selected the following sythases: RpaI, BraI, RhlI, BjaI, EsaI, LuxI, AubI, LasI, and CerI (Fig 2). This set represents HSLs with chain lengths from 3 to 18 carbons and modifications including phenol, phenyl, carbonyl, and methyl groups. We included LuxI as it is the most commonly used network and the cognate sender to our LuxR receiver device. While the inclusion of EsaI may seem redundant as they both produce N-(3-oxo-hexanoyl)-l-homoserine lactone (3-oxo-C6-HSL) as their major HSL, Gould *et al*. showed that EsaI only produces 3-oxo-C6-HSL in *E*. *coli* and therefore may have no secondary HSL products [27]. We also included SinI despite its promiscuity because of the unique HSLs it synthesizes, including the unsaturated 3-oxo-7,8-cis-C16-HSL and the long chain N-octadecanoyl-l-homoserine lactone (C18-HSL) [35].

**Fig 3:**
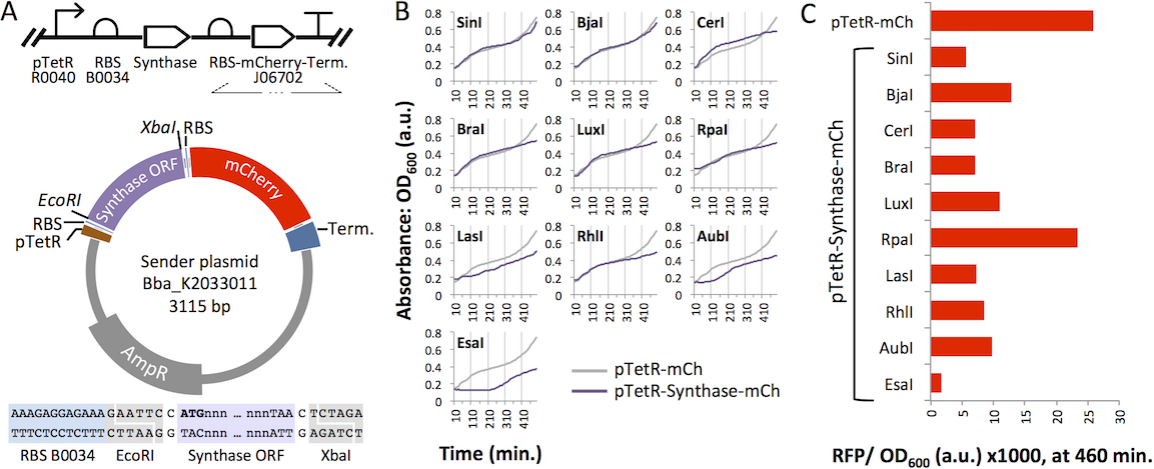
Sender plasmids expressed in *Escherichia coli* BL21. (A) The modular Sender vector allows facile cloning of any *EcoRI*, *XbaI*-flanked synthase open reading frame (ORF) and co-expression with mCherry (mCh) from a single bicistronic mRNA. The vector is represented as SBOL [44] glyphs (top) and a scaled circular map (bottom). (B) Optical density (OD) readings for *E*. *coli* cultures expressing each of the Sender plasmids. (C) mCherry expression is shown as end-point RFP signal normalized to OD_600_ after an 8 hour growth period.

We obtained synthesized DNA based on the publicly available sequences for each synthase open reading frame (ORF) (Supporting Information). We designed and constructed a plasmid for simple directional cloning and high expression of HSL synthase homologues in *E*. *coli* (Fig 3A). The Sender vector (Bba_K2033011) includes a strong constitutive promoter (pTetR), a strong ribosome binding site (B0034), and high-copy number origin of replication (pUC19-derived pMB1, 100–300 copies per cell) for maximal protein expression and HSL production, with the option to switch to an inducible expression approach (in a chassis that expresses the TetR protein) [43]. The vector also carries a fluorescent protein ORF, mCherry, for pTetR-driven bicistronic expression allowing us to visually confirm consistent expression between batches of Sender cultures (Fig 3A). Sender plasmids are designed such that the synthase and mCherry proteins are expressed from a single bicistronic mRNA transcript, and the synthase ORF appears upstream of the mCherry ORF. Therefore, expression of mCherry indicates the presence of full-length synthase mRNA. To confirm the co-expression of mCherry and each synthase protein, we carried out denaturing polyacrylamide gel electrophoresis (PAGE) followed by staining with coomassie dye R-250. We analyzed lysates from cells transformed with an mCherry-only plasmid (pTetR-mCh) or a Sender plasmid (S1 Fig). Bands corresponding to the expected molecular weight of mCherry were visible in all samples except for the negative controls (Control-EGFP and untransformed cells). Unique bands in LuxI, CerI, AubI, and LasI Sender lysates corresponded to the predicted molecular weights of these synthases. However, the most predominant unique bands for the other Senders appeared to have larger (SinI) or smaller (RhlI, RpaI, BraI, EsaI, BjaI) molecular weights than expected. In these cases, the presence of sub-optimal codons may have compromised expression of full-length proteins at high levels in *E*. *coli*.

We carried out fluorescence plate reader assays to monitor mCherry expression levels in BL21 *E*. *coli* that were transformed with one of each Sender variant. The mCherry-only plasmid (pTetR-mCh, without a synthase ORF) was used as a positive control for RFP signal. While most of the synthase-expressing cultures showed growth that tracked closely with the pTetR-mCh control, the lag in growth up to 110 or 260 minutes for and LasI, AubI, and EsaI suggests synthase-specific metabolic burden or toxicity in these three cultures (Fig 3B). Red fluorescence protein (RFP) signal values normalized by absorbance (OD_600_) indicated that mCherry expression was roughly 12% to 50% that of the control, with the exception of RpaI. Incomplete transcription or less efficient expression from the larger pTetR-Synthase-mCherry plasmids (585–714 additional bp) may account for lower mCherry signals. An unidentified cryptic promoter within the *rpaI* ORF could drive enhanced mCherry expression. Overall, these results validate the production of mRNA transcripts containing both the HSL synthase and the mCherry ORF and show that mCherry expression increases over time. We submitted to the iGEM Registry of Standard Parts sequences that were not previously represented in this public collection (S1 Table and S1 Appendix).

### Induction of a LuxR receiver device with synthases from the Sender library

**Fig 4:**
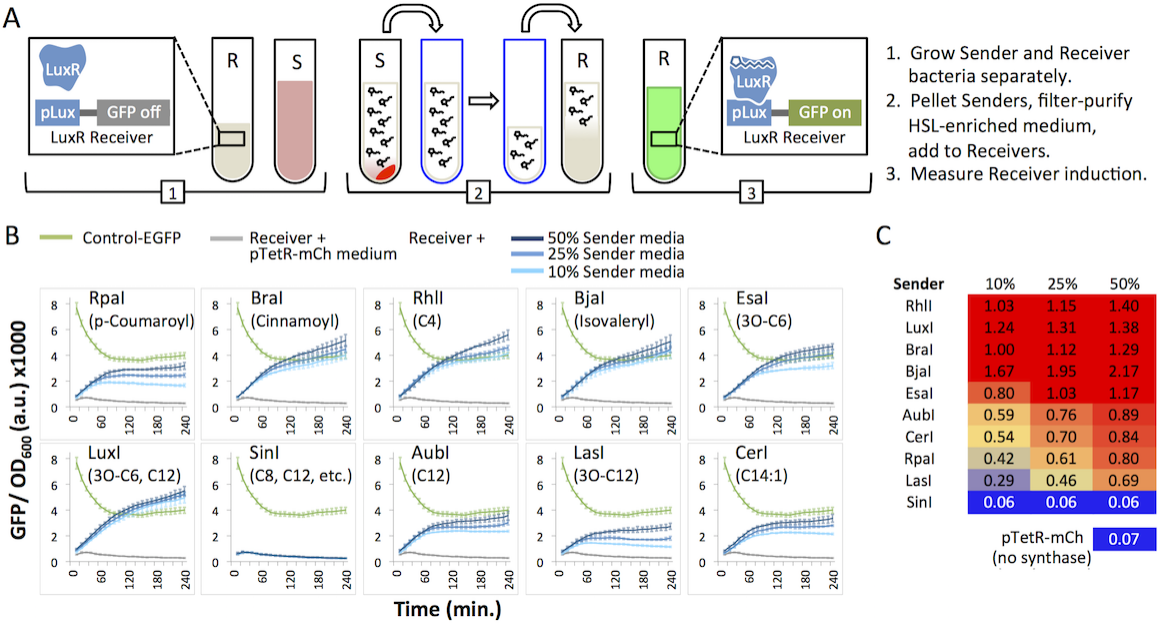
Induction of the LuxR receiver with HSL-enriched media from Senders. (A) The cartoon illustrates the experimental approach for Receiver inductions. Sender (S) culture medium is separated from pelleted Sender cells, filtered, and diluted (see Table 1), and used to induce Receiver cells. (B) GFP/OD_600_ of Sender-media-treated LuxR receiver cells. “Control-EGFP” bacteria carry a plasmid that constitutively expresses EGFP from a pLac promoter. EGFP and optical density (OD_600_) are measured every 10 minutes for 240 minutes (4 hours). Graphs show means of triplicate wells (bars, standard deviation). (C) The heat map shows GFP/ OD_600_ values after 240 min of induction, normalized to the GFP/ OD_600_ value for Control-EGFP.

In order to measure Sender functionality, we induced a LuxR receiver device with HSL-enriched, cell-free media from Sender cultures. The promiscuity of Lux signal-sensing is well established [11] and it is often used to test for the general presence of HSLs [16]. The BioBrick plasmid BBa_F2620 constitutively expresses the LuxR regulator protein and contains the inducible pLux promoter [11]. We cloned EGFP (BBa_E0240) downstream of pLux so that upon addition of a strong ligand (HSL), LuxR would bind the pLux promoter and induce EGFP expression. We transformed a common lab chassis, *E*. *coli* BL21 with our LuxR receiver device, F2620_EGFP, or with each of the Sender plasmids. We grew the Senders and Receivers in separate liquid cultures, collected and filtered HSL-enriched media from Sender cultures, or mock-enriched medium from mCherry control bacteria, and treated Receiver cells with the filtered media (Fig 4A, Table 1). EGFP signal and OD_600_ were measured over time (4 hours) for each sample using a plate reader.

**Table 1:** Preparation of receiver induction experiments.

Sample	Sender medium (μL)	Mock-enriched medium (μL)	Total	Final
Control-EGFP + 50% pTetR-mCh medium	0	150	150	300
Receiver + 50% pTetR-mCh medium	0	150	150	300
Receiver + 10% Sender medium	30	120	150	300
Receiver + 25% Sender medium	75	75	150	300
Receiver + 50% Sender medium	150	0	150	300

The total volume of mixed media (Total, 150 μL) was added to 150 μL of Receiver or Control-EGFP liquid culture for a final volume of 300 μL.

We considered results reported by Canton et al [45] as a model to predict and interpret the results from our Synthase-driven induction experiments. Their experiment established the range of responses and relative sensitivity of BBa_F2620 (LuxR) to known concentrations of homogenous HSL solutions. If we assume equal concentrations of homogenous HSL production from each *E*. *coli*-expressed synthase, we can hypothesize that the media will show relatively strong to weak (or no) stimulation of pLux-EGFP in the following order: LuxI (3-oxo-C6, C12) or EsaI (3-oxo-C6), SinI (C8, C12, and others), AubI (C12), RhlI (C4). We cannot make a prediction for the synthases that are not represented in the Canton et al experiment: RpaI (p-Coumaroyl), BraI (Cinnamoyl), BjaI (Isovaleryl), CerI (C14:1), and LasI (3-oxo-C12). Our prediction is confounded by the possibility that transgenically expressed non-native synthases generate a mixture of active and inactive products at varying concentrations, which can be difficult to analyze quantitatively. Therefore, pLux-EGFP induction using synthetic HSLs has limited applicability for cell-based inducers.

We observed EGFP signal above background (Receiver plus mock-enriched medium) for all but one of our Senders, SinI (Fig 4B). We can conclude from these data that nine synthases expressed bioactive, cell membrane permeable HSLs in *E*. *coli* BL21. Although the observed bands from the PAGE analysis (S1 Fig) did not correspond to the expected molecular weights for RhlI, RpaI, BraI, EsaI, BjaI, the pLux-EGFP induction results suggest the synthase proteins are functional. We also measured signal from a constitutively-expressed pLac-EGFP (called “Control-EGFP” here) to establish a threshold for full activation. Prior to the experiment, Receiver cells (pLux-EGFP) and Control-EGFP cells were grown to an OD_600_ of 2.0 (stationary phase), then diluted with HSL-enriched or mock-enriched (pTetR-mCh) media. Control-EGFP cells showed high initial total EGFP signal that increased (~3-fold) over time (S2 Fig). The normalized signal (GFP/OD_600_, Fig 4B) decreased over time and reached steady state after ~90 min, which may be a consequence of decreasing Control-EGFP plasmid and EGFP molecules per cell as the cells divide. At 240 minutes (6 hours) post-induction, Receivers treated with any of the three concentrations of Sender media (10%, 25%, 50%) from RhlI, LuxI, BraI, and BjaI generated EGFP signal that reached Control-EGFP levels (Fig 4C, values ≥ 1.0). For EsaI, 25% and 50% Sender medium was sufficient to induce full activation of EGFP. EsaI and LuxI both produce 3-oxo-C6-HSL in their native organisms [28,38] and *E*. *coli* [27] which strongly induces LuxR [45]. We expect RhlI to produce mostly N-butyryl-l-homoserine lactone (C4-HSL) which Canton *et al*. found to only induce this LuxR receiver at high concentrations [45]. However, Ortori *et al*. found that *Pseudomonas aeruginosa*, when mutated to only express RhlI, produced small amounts of N-hexanoyl-l-homoserine lactone (C6-HSL) and 3-oxo-C6-HSL [46]. The degree of LuxR response to RhlI that we observed is more consistent with this HSL profile and suggests that in *E*. *coli* BL21, RhlI produces C6-HSL and/or 3-oxo-C6-HSL. This result demonstrates the importance of testing synthases in context.

LasI, AubI, CerI, and RpaI showed induction above background at all dilutions, however, pLux-EGFP did not become fully activated (Fig 4B). These synthases are expected to produce HSLs with longer chains (Fig 2). LuxR could interact poorly with these HSL ligands or the synthases could have low activity in our chassis.

### Characterization of sender induction in solid agar cultures

**Fig 5:**
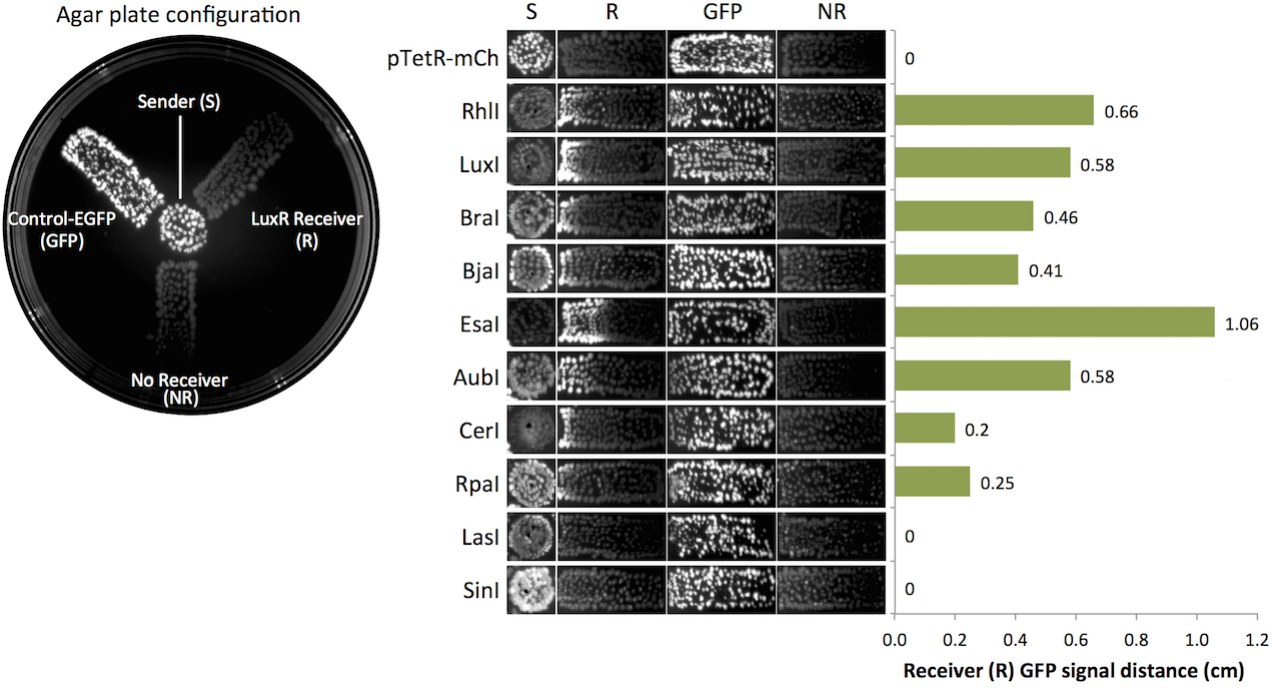
LuxR induction in solid agar cultures. The leftmost image shows the layout used to determine induction distance across agar. Sender bacteria were spotted in the center. Bacteria transformed with Control-EGFP (GFP), receiver negative (NR), or LuxR receiver (R) plasmids were spotted within rectangular areas that radiated away from the center. Induction distance in centimeters is shown for each of the ten Senders tested.

We next determined the behavior of our sender devices in a different context: spatially separated from the receiver device in solid agar cultures. We plated each Sender in the center of a 10 cm agar plate and applied Receiver bacteria across an area spanning up to 3.0 cm from the Sender spot (Fig 5). After 16 hours of growth we measured the induction distance, the length from the center where the Sender was plated to the edge of the detectable GFP signal in the area were LuxR Receiver cells were grown (Fig 5). The strongest inducers of LuxR in liquid culture, RhlI, LuxI, BraI, BjaI, and EsaI (Fig 4B), all induced LuxR on the agar plate. RpaI and CerI pLux-EGFP induction values were lower than those for the five strongest inducers in both experiments. Also consistent with the liquid induction experiment, SinI showed no induction of LuxR on the agar plate. In certain cases however, the relative performances of synthases were inconsistent in liquid versus agar cultures. For instance, EsaI induced pLux-EGFP over the longest distance overall, and almost two-fold the distance for the cognate synthase LuxI. However, EsaI did not induce the Receiver as strongly as LuxI in the liquid culture experiments. While LuxR responded to to LasI HSL-enriched liquid medium in a dose-dependent manner (Fig 4B), we detected no EGFP signal over background on the agar plate (Fig 5). Finally, the maximum value of AubI-induced pLux-EGFP (50% enriched medium) was roughly half that of the strongest inducers (Fig 4C), whereas the distance of AubI-induced EGFP signal in the agar experiment (0.58 cm) fell within the range of values for the same inducers (BjaI, 0.41; RhlI, 0.66 cm) (Fig 5). Inconsistencies between the liquid and agar culture experiments might be due to HSL-specific diffusion limits in agar where there is no mechanical mixing, or differences in the stability of the HSLs in each medium. Furthermore, the agar experiment exposed Receiver bacteria to continual expression of each synthase from a growing population of Sender cells. In contrast, the fixed amount of available HSLs in the liquid culture experiments would be more affected by decay. Possible differences in enzyme-specific HSL production rates over the 16-hour period of plate incubation might account for the observed difference between LuxI and EsaI, which are expected to produce the same HSL. Overall, our results underscore the importance of testing devices in the chassis and context relevant to the application of interest.

### Comparison of conventional disposal methods: Autoclaving is more effective than bleach for quenching HSL activity

We next tested whether standard hazardous waste treatment methods, bleaching and autoclaving, are sufficient to inactivate media containing HSLs from four Senders, LuxI, LasI, RpaI, and CerI [47]. These Senders span the range of LuxR-activation strengths we observed in the liquid culture experiments. Borchardt et al. showed that 3-oxo-containing HSLs are sensitive to oxidation in bleach solution, while other HSLs are resistant [48]. Therefore, we expected bleach to specifically quench the activity of HSL-enriched media from LuxI (3-oxo-C6-HSL) and LasI (3-oxo-C12-HSL).

**Fig 6:**
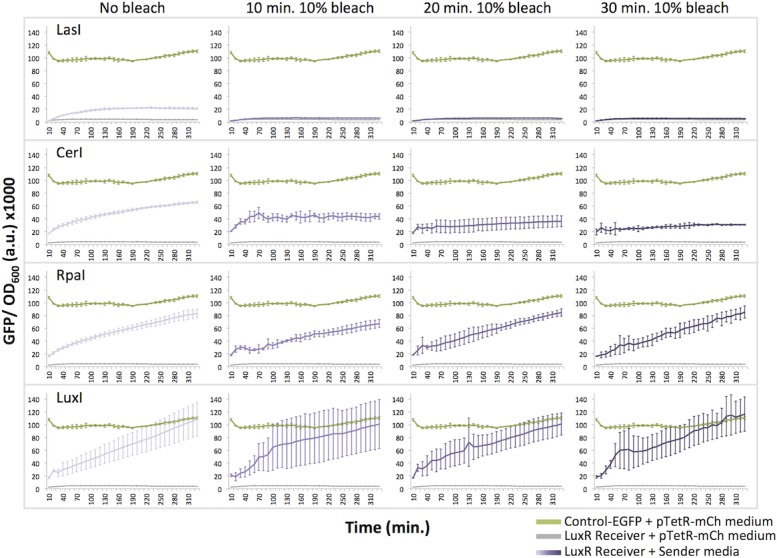
Bleach is not sufficient to deactivate media containing homoserine lactones (HSLs). Induction of the LuxR receiver device with untreated or treated media from LasI, CerI, RpaI, and LuxI Sender cultures. Treated cultures were incubated for 10 min, 20 min or 30 min at a final concentration of 10% bleach.

We added bleach to a final concentration of 10% to overnight cultures of Sender-transformed *E*. *coli* BL21 for 10, 20, or 30 minutes. HSLs were extracted, added to liquid cultures of LuxR, and EGFP production was measured over time (330 minutes, or 5.5 hours). Consistent with the findings from Borchardt et al., we observed that bleach treatment eliminated the activity of the LasI-HSL-enriched medium, while media from CerI and RpaI remained active (Fig 6). Intriguingly, LuxI-HSL-enriched medium showed no reduction in activity, which does not agree with the idea that oxidation destroys 3-oxo-C6-HSL activity. This result suggests the presence of bleach-resistant secondary HSLs or that the sterilization conditions used here are insufficient to destroy the activity of high concentrations of 3-oxo-C6-HSL. Overall, we conclude that treatment with 10% bleach for up to 30 minutes at room temperature is not a sufficient approach for quenching all HSL bioactivity.

**Fig 7:**
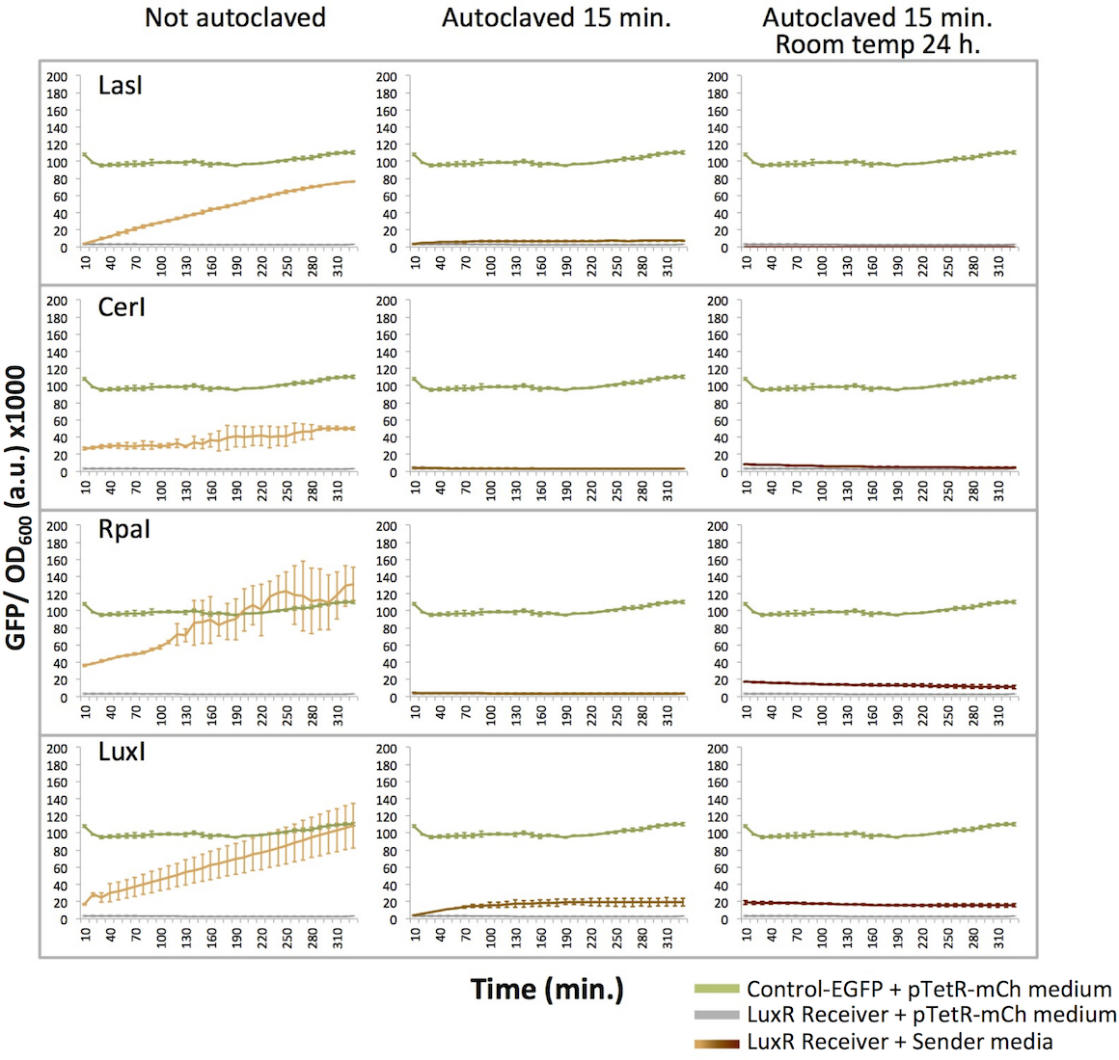
Autoclaving deactivates media containing homoserine lactones (HSLs). Induction of the LuxR receiver device with untreated or treated media from LasI, CerI, RpaI, and LuxI Sender cultures. Treated cultures were autoclaved for 15 min. and used to induce LuxR receiver cultures immediately or after a 24 h incubation at room temperature.

To determine whether autoclave treatment is effective for deactivating HSLs, overnight cultures of *E*. *coli* BL21 transformed with the four Sender plasmids were autoclaved for 15 min at 121°C, 15 psi. HSLs were extracted and added to liquid cultures of LuxR immediately after autoclaving or after a 24 hour incubation at room temperature to represent the typical practice of storage between autoclaving and final disposal. For both immediate and delayed induction, autoclave treatment sufficiently inactivated HSLs produced by RpaI, CerI, and LasI Senders (Fig 7). The autoclaved LuxI medium showed some induction above background. However it is substantially reduced compared to untreated LuxI medium. We conclude that autoclaving is the more effective treatment to deactivate media containing HSLs prior to disposal.

## Discussion

This study expands the quorum sensing toolbox by characterizing a library of ten HSL sender devices in a commonly used lab chassis, *E*. *coli* BL21 in two contexts, liquid medium and solid agar cultures. Using a receiver device, LuxR, we confirmed functionality for nine of ten Senders. By testing first in the chassis (*E*. *coli*) of interest at multiple dilutions, one can quickly gather data on how devices will behave in an *in vivo* circuit. Previous studies have demonstrated the orthogonality of LasI/LuxR in *E*. *coli* and RpaI/LuxR in *Salmonella* Typhimurium and implemented circuits without crosstalk [13,18]. In our study, LasI and RpaI did not induce full activation (compared to Control-EGFP) at low concentrations, and approached full activation (~80%) at the highest concentration of HSL-enriched media. Assuming that synthase expression can be tuned by using promoters and RBSs of different strengths, or origins of replication with different copy numbers, an engineer could exploit the varying sensitivity of a Receiver (e.g. LuxR) to mitigate crosstalk between parallel quorum sensing pathways.

Furthermore, synthases are known to produce different HSL profiles when expressed in non-native bacteria [27,46]. An HSL profile determined in one experiment (e.g., via mass spectroscopy or HPLC) may not be sufficient to predict the impact of a transgenic synthase on a Receiver. In our study, we selected synthases based on characterized HSL profiles, but determined their bioactivity using live cultures. Our approach does not require mass spectrometry, which can be expensive and inaccessible to certain researchers such as small labs, teaching labs, and iGEM teams.

Our work also demonstrates the importance of testing quorum sensing networks in the context of interest. Liquid culture induction results did not predict relative induction distances solid agar cultures. We observed a wide range of induction distances for synthases that induced similarly in liquid cultures. LasI, which induced LuxR at high dilutions in liquid culture, did not induce LuxR on the agar plate. These data exemplify the importance of testing networks in context and provide researchers with data to inform design of agar-based devices using Sender and Receiver pairs that show high levels of crosstalk in other contexts. Interestingly, we did not see a gradient of induction on the agar plate inductions. This is consistent with the behavior of the edge detection device built by Tabor *et al*. and suggests other Senders could be used in this kind of device to create edges of varying thicknesses [49].

Finally, our work underscores the need for more careful consideration of the disposal of HSL-enriched media. Risks associated with cell-generated metabolites, i.e. HSLs, have not been addressed as thoroughly as the risks of environmental contamination by live cells [50]. In 2001, Borchardt et al. reported that acetylated homoserine lactones that lack the 3-oxo group failed to react with oxidized halogens (e.g., sodium hypochlorite, or bleach) and retained their ability to stimulate a QS-regulated gene in live cells while HSLs containing the 3-oxo group were effectively deactivated [48]. Consistent with their results, we found that cultures treated with bleach were still able to induce the LuxR receiver. We discovered that autoclaving sufficiently reduced bioactivity for all HSL-enriched media tested in our study. Further work should analyze a wider range of HSL to identify a universal sterilization strategy. We recommend that scientists who regularly use HSL-producing cultures perform their own bioactivity tests or autoclave their cultures before disposal. It is important to note that while we observed induction with bleached cultures in the lab, this does not suggest that environmental bacteria are being induced by disposed media. Our culture volumes were on the order of 1 mL to 100 mL which are immediately diluted by many orders of magnitude upon disposal. We have not provided evidence that additional safety precautions are necessary for using quorum sensing networks that are expressed in lab safe bacterial strains, grown in small volumes. Gene synthesis companies do not consider quorum sensing genes as hazardous when they are derived from a non-pathogenic bacterium. Many quorum sensing synthases are available in the iGEM registry and other repositories. We recommend that these organizations consider providing additional information on proper disposal.

In conclusion, our work adds to the rich, growing body of knowledge to inform design principles and best practices for the use of HSL-modulated quorum sensing networks in engineered circuits [11,23]. A series of recent publications has greatly expanded the number of HSL Receiver-type devices available to genetic engineers [23,51–53]. Our work to build and characterize a library of diverse senders complements these efforts to enable researchers to construct more complex networks with minimal crosstalk, and provides an approach to identify functionally orthogonal sender-receiver pairs for parallel computation in different cell culturing conditions.

## Methods

### Plasmid constructs

DNA digests included 1 μL (each) FastDigest enzymes and 1x universal buffer from Thermo Fisher Scientific and ~2–4 μg DNA in a final volume of 30 μL, incubated at the lowest appropriate temperature for 10 min. Ligations included a 2:1 molar ratio of insert:vector, 1 μL T4 DNA ligase (New England Biolabs #M0202), and 1x Rapid ligation buffer (Roche #11635379001) in a final volume of 10–15 μL incubated at room temperature for 5 min. ***Inducible LuxR receiver***: BBa_F2620 (iGEM Headquarters) contains the promoter pTetR controlling expression of the LuxR regulator and the LuxR-HSL regulated promoter pLuxR. A *XbaI-PstI* fragment (RBS-EGFP-2xTerminator) from BBa_E0240 was ligated downstream of pLuxR into a SpeI-PstI-linearized BBa_F2620 vector. ***Modular Sender Vector***: pTetR (BBa_R0040) was inserted upstream of an RBS (BBa_B0034) in pSB1A3 using BioBrick assembly [54]. pTetR-RBS was amplified with High Fidelity Phusion PCR (Thermo Fisher Scientific #F530, manufacturer’s instructions) using primers 5’-ctaggaatttagtcttc**tccctatcagtgata** (forward) and 5’-actagtc[tctaga]agcggccgc[gaattc]**tttctcctctttctc** (reverse), then double digested with BbsI (to generate an *EcoRI*-compatible overhang 5’-aatt) and SpeI. Similarly, a RBS-mCherry-2xTerminator fragment was amplified from BBa_J06702 (iGEM Headquarters) using primers 5’-actagt**aaagaggagaaatac** (forward) and 5’-ctagctgcaga**tataaacgcagaaag** (reverse), then double digested with SpeI and PstI. Both fragments were ligated into a EcoRI-PstI-linearized pSB1A3 vector. For the preceding primer sequences: underlined text, restriction sites for cloning; bold text, template binding sequence; brackets, *EcoRI* and *XbaI* sites used for synthase ORF inserts (described below). Complete, annotated sequences for all Sender plasmids and the Receiver plasmid are available at the Haynes Lab Benchling website: https://benchling.com/hayneslab/f_/UWzcp3nk-quorum-sensing-collection. In addition, all Sender plasmids are available through the iGEM Registry of Standard Parts (parts.igem.org, BBa_K1421006, BBa_K2033004, BBa_C0170, BBa_K2033002, BBa_K1670004, BBa_C0161, BBa_K2033008, BBa_K2033000, BBa_C0078, and BBa_K2033006).

### Cloning of HSL synthase homologues

The coding regions for the following HSL synthases were synthesized as double-stranded oligos (IDT) with an *EcoRI* binding site upstream and a *XbaI* cut site downstream: AubI, BjaI, BraI, CerI, EsaI, LasI, LuxI, RhlI, RpaI, and SinI. The Modular Sender Vector and each HSL synthase oligo were cut with EcoRI and XbaI (Thermo Fisher Scientific) and ligated using T4 ligase (New England Biolabs). Plasmids containing synthases not already in the iGEM registry were submitted with the following identification keys: AubI BBa_K2033000, BjaI BBa_K2033002, BraI BBa_K2033004, CerI BBa_K2033006, SinI BBa_K2033008.

### Sender media preparation

Cells transformed with Sender plasmids or an mCherry (no synthase) plasmid (pTetR-mCh) were grown as colonies, and a single colony from each was used to inoculate 3 ml of LB with 5μg/ml ampicillin (VWR). Cultures were grown for 16 h at 37°C with shaking. Sender and mCherry cultures were spun at 4500g for 5 min. Supernatants were passed through 0.22 μm nylon filters (VWR International) to remove any remaining cells.

### Microwell plate reader assays

Single colonies of *E*. *coli* BL21 cells transformed with Receiver (F2620-EGFP) or Control-EGFP plasmid (pTrc99A vector expressing EGFP from the pLac promoter) were inoculated in 3 mL of LB broth [25g Acros LB broth Lennox granules (Sigma) in 1000 mL water] with 5 μg/mL ampicillin (VWR) and grown for 16 h at 37°C with shaking. Each of culture was used to inoculate a new culture with a starting absorbance (OD_600_) of 0.05, and grown at 37°C with shaking to a final OD_600_ of 0.8. Cultures were spun at 4500 xg for 5 min. Supernatants were discarded and cells were resuspended in fresh LB with ampicillin (OD_600_ = 0.8). Corning Black Costar Clear Bottom 96 Well Plates (Fisher Scientific) were loaded with 150 μL Receiver or Control-EGFP of a total volume of 300 μL per well with a final OD_600_ of 0.4. Varying concentrations of Sender and mock-enriched (pTetR-mCh) media were used to ensure the same total volume of spent medium per well (Table 1). Plates were analyzed on a Biotek Synergy H1 Microplate Reader, measuring red fluorescence (580 nm to 610 nm), green fluorescence (485 nm to 515 nm), and OD_600_ every 10 min for 8h at 37°C with shaking. Automatic gain adjustment was set to scale to the lowest detected well values for each measurement. Mean values and standard deviations of GFP signal / OD_600_ (a.u.) were calculated for triplicate wells at each 10 minute time point. Graphs and heat maps were generated in Microsoft Excel 2016.

### Agar plate inductions

Cultures of *E*. *coli* BL21 (New England Biolabs) transformed with Sender plasmids, a Receiver plasmid, a negative receiver plasmid, and a GFP positive plasmid were grown in 3 mL of LB with 5 ug/mL ampicillin for 16 hours at 37°C and shaking at 220 rpm. Bacterial culture was subsequently spread onto LB agar supplemented with 5 ug/mL ampicillin with sterile disposable plastic micropipette tip, such that a central spot of Sender culture would evenly diffuse towards proximal Receiver and Control-EGFP positive control cultures. Plates were grown for 16 hours at 37°C. Images were acquired with a Pxi4 imager under ultraviolet light, saved at 300 dpi resolution, and analyzed using ImageJ software. Edges of fluorescence-positive areas were determined as the area in which the raw integrated density within a window of 50×100 pixels was equal to that of GFP-minus cells. Induction distances were determined as the shortest distance (straight line) from the Sender-proximal edge of the Receiver cells to the edge of the fluorescence positive area.

### Bleach and autoclave treatment of sender media

HSL-enriched media (prepared as described above) from all Senders were treated with bleach (sodium hypochlorite solution) at a final concentration of 10%. Bleach treatment was performed by adding 1 volume of bleach (Genesee Scientific) to 10 volumes of filter-purified, HSL-enriched medium. Samples were incubated at room temperature for 10, 20 or 30 min. At each time point, 1 mL of solution was removed and added to an equal volume of ethyl acetate for HSLs extraction. The extraction solution was incubated for 1 minute at room temperature while shaking, and then allowed to phase separate for 10 min at room temperature. The organic phase was collected and subjected to rotary evaporation. The dried samples were resuspended with 1 mL of sterile, double distilled H_2_O. A control sample of Sender medium without bleach was also extracted at each time point. For autoclave treatments, 1 mL of HSL-enriched medium was sealed in a glass round-bottom tube and autoclaved for 15 min at 121°C and 15 psi (BetaStar 26 x 26 x 39 Automatic Vertical Sliding Autoclave). After cooling, samples were extracted as described above. Receiver inductions were carried out on treated and extracted HSL-enriched media as described above.

## Supporting information

S1 Appendix Modular sender DNA sequences in FASTA format. The following list includes sequences of the open reading frames for the ten synthase proteins used in this study. The FASTA header includes the symbol of the synthase (e.g. RpaI), followed by an abbreviation for the species of origin (e.g. R_palustris, *R*. *palustris*), the NCBI Accession number (e.g. WP_011155888), and length in bp (e.g. 657bp). Start (ATG) and stop codons are shown in bold font.
(DOCX)

S1 Table Relevant iGEM Registry IDs. “Antiquity” indicates that no specific team is known to have contributed the DNA sequence to the Registry. Entries can be accessed at http://parts.igem.org/
(DOCX)

S1 Fig PAGE analysis of lysates from sender transformants. Sender cells were grown overnight in liquid media, pelleted, and lysed before electrophoresis of whole protein lysate for size analysis. Sender cell lysates were electrophoresed on a 4–12% Bis-Tris polyacrylamide gel at 200 V for 50 minutes in 1x MOPS buffer. Gels were stained for 1 hour with coomassie dye R-250 (Imperial Protein Stain, Thermo fisher #24615) for one hour, followed by two hours of destaining in water. The first lane of each gel is a standard ladder (Invitrogen #LC5800); 30, 20, and 15 kDa bands are marked. Expected protein sizes were calculated using CLC Main Workbench ver. 8.0.1 Protein Report tool. Red arrows indicate the mCherry protein (expected size 26.7 kDa). White arrows indicate expected synthase protein bands. (A) A comparison of lysates from Control-EGFP cells and LuxI Sender (pTetR-LuxI-mCherry) cells shows bands that correspond with the expected sizes for mCherry (26.7 kDa) and LuxI (22.3 kDa) only in the LuxI Sender lysate. The bottom image shows band intensity traces generated by the ImageJ (ver. 1.51s, Macintosh) Gel Analyzer tool. (B) Lysates from the other nine Senders were subjected to PAGE in two trials (top gel, BjaI and LasI 2 μL per lane, others 5 μL; bottom gel, 2 μL lysate per lane). (C) Band intensity traces for the gels shown in B. Data from the lanes in gel 2 (bottom) were arranged to match the order of synthases for gel 1 (top). The trace from a non-transformed control (blue) is overlaid with pTetR-mCherry (red) to distinguish the mCherry peak. For other lanes, the pTetR-mCherry trace is overlaid with Sender (pTetR-Synthase-mCherry) traces (grey).
(DOCX)

S2 Fig EGFP expression from Control-EGFP over time. The plots show the unnormalized data from Fig 4: Control-GFP cells grown in mock-enriched medium (from pTetR-mCh cells); left, Absorbance (OD_600_) over time; right, unnormalized GFP signal over time. EGFP and optical density (OD_600_) were measured every 10 minutes for 240 minutes (4 hours). Graphs show means of triplicate wells (bars, standard deviation). We observed that the relative GFP values (signal divided by OD_600_) decreased over time (Fig 4B). However, the raw data show that total GFP expression increases over time during culture growth.
(DOCX)

## References

[1] Ruby EG, Nealson KH. Symbiotic Association of Photobacterium Fischeri with the Marine Luminous Fish Monocentris Japonica: A Model Of Symbiosis Based on Bacterial Studies. Biol Bull. 1976;151: 574–586. 10.2307/1540507 1016667

[2] Eberl L. N-acyl homoserinelactone-mediated gene regulation in gram-negative bacteria. Syst Appl Microbiol. Urban & Fischer Verlag; 1999;22: 493–506. 10.1016/S0723-2020(99)80001-0 10794136

[3] Kleerebezem M, Quadri LE, Kuipers OP, de Vos WM. Quorum sensing by peptide pheromones and two-component signal-transduction systems in Gram-positive bacteria. Mol Microbiol. 1997;24: 895–904. 9219998 10.1046/j.1365-2958.1997.4251782.x

[4] Fuqua C, Winans SC, Greenberg EP. Census and consensus in bacterial ecosystems: the LuxR-LuxI family of quorum-sensing transcriptional regulators. Annu Rev Microbiol. 1996;50: 727–751. 10.1146/annurev.micro.50.1.727 8905097

[5] Williams P, Winzer K, Chan WC, Camara M. Look who’s talking: communication and quorum sensing in the bacterial world. Philos Trans R Soc Lond B Biol Sci. 2007;362: 1119–1134. 10.1098/rstb.2007.2039 17360280 PMC2435577

[6] Dickschat JS. Quorum sensing and bacterial biofilms. Nat Prod Rep. 2010;27: 343–369. 10.1039/b804469b 20179876

[7] Case RJ, Labbate M, Kjelleberg S. AHL-driven quorum-sensing circuits: their frequency and function among the Proteobacteria. ISME J. 2008;2: 345–349. 10.1038/ismej.2008.13 18273067

[8] Kimura N. Metagenomic approaches to understanding phylogenetic diversity in quorum sensing. Virulence. 2014;5: 433–442. 10.4161/viru.27850 24429899 PMC3979871

[9] Engebrecht J, Silverman M. Identification of genes and gene products necessary for bacterial bioluminescence. Proc Natl Acad Sci U S A. 1984;81: 4154–4158. 6377310 10.1073/pnas.81.13.4154 PMC345387

[10] Kaplan HB, Greenberg EP. Overproduction and purification of the luxR gene product: Transcriptional activator of the Vibrio fischeri luminescence system. Proc Natl Acad Sci U S A. 1987;84: 6639–6643. 16578817 10.1073/pnas.84.19.6639 PMC299138

[11] Davis RM, Muller RY, Haynes KA. Can the Natural Diversity of Quorum-Sensing Advance Synthetic Biology? Frontiers in Bioengineering and Biotechnology. 2015;3: 1–10. 10.3389/fbioe.2015.00001 25806368 PMC4354409

[12] Anderson JC, Clarke EJ, Arkin AP, Voigt C a. Environmentally controlled invasion of cancer cells by engineered bacteria. J Mol Biol. 2006;355: 619–627. 10.1016/j.jmb.2005.10.076 16330045

[13] Balagaddé FK, Song H, Ozaki J, Collins CH, Barnet M, Arnold FH, et al A synthetic Escherichia coli predator-prey ecosystem. Mol Syst Biol. 2008;4 10.1038/msb.2008.24 18414488 PMC2387235

[14] You L, Cox RS, Weiss R, Arnold FH. Programmed population control by cell–cell communication and regulated killing. Nature. 2004;428: 868–871. 10.1038/nature02491 15064770

[15] Ji W, Shi H, Zhang H, Sun R, Xi J, Wen D, et al A formalized design process for bacterial consortia that perform logic computing. PLoS One. 2013;8: e57482 10.1371/journal.pone.0057482 23468999 PMC3585339

[16] Wang B, Barahona M, Buck M. A modular cell-based biosensor using engineered genetic logic circuits to detect and integrate multiple environmental signals. Biosens Bioelectron. Elsevier; 2013;40: 368–376. 10.1016/j.bios.2012.08.011 22981411 PMC3507625

[17] Tamsir A, Tabor JJ, Voigt CA. Robust multicellular computing using genetically encoded NOR gates and chemical “wires.” Nature. 2011;469: 212–215. 10.1038/nature09565 21150903 PMC3904220

[18] Scott SR, Din MO, Bittihn P, Xiong L, Tsimring LS, Hasty J. A stabilized microbial ecosystem of self-limiting bacteria using synthetic quorum-regulated lysis. Nature Microbiology. 2017;2: 17083 10.1038/nmicrobiol.2017.83 28604679 PMC5603288

[19] von Bodman SB, Ball JK, Faini MA, Herrera CM, Minogue TD, Urbanowski ML, et al The quorum sensing negative regulators EsaR and ExpR(Ecc), homologues within the LuxR family, retain the ability to function as activators of transcription. J Bacteriol. 2003;185: 7001–7007. 10.1128/JB.185.23.7001-7007.2003 14617666 PMC262718

[20] Saeidi N, Wong CK, Lo T-M, Nguyen HX, Ling H, Leong SSJ, et al Engineering microbes to sense and eradicate Pseudomonas aeruginosa, a human pathogen. Mol Syst Biol. 2011;7: 521 10.1038/msb.2011.55 21847113 PMC3202794

[21] Shong J, Huang Y-M, Bystroff C, Collins CH. Directed evolution of the quorum-sensing regulator EsaR for increased signal sensitivity. ACS Chem Biol. 2013;8: 789–795. 10.1021/cb3006402 23363022 PMC4478592

[22] Wu F, Menn DJ, Wang X. Quorum-sensing crosstalk-driven synthetic circuits: from unimodality to trimodality. Chem Biol. 2014;21: 1629–1638. 10.1016/j.chembiol.2014.10.008 25455858 PMC4272683

[23] Scott SR, Hasty J. Quorum Sensing Communication Modules for Microbial Consortia. ACS Synth Biol. 2016;5: 969–977. 10.1021/acssynbio.5b00286 27172092 PMC5603278

[24] Byers DM, Gong H. Acyl carrier protein: structure-function relationships in a conserved multifunctional protein family. Biochem Cell Biol. 2007;85: 649–662. 10.1139/o07-109 18059524

[25] Heath RJ, Rock CO. Regulation of Fatty Acid Elongation and Initiation by Acyl-Acyl Carrier Protein in Escherichia coli. J Biol Chem. 1996;271: 1833–1836. 8567624 10.1074/jbc.271.4.1833

[26] Rock CO, Jackowski S. Regulation of phospholipid synthesis in Escherichia coli. Composition of the acyl-acyl carrier protein pool in vivo. J Biol Chem. 1982;257: 10759–10765. 6809756

[27] Gould T a., Herman J, Krank J, Murphy RC, Churchill ME a. Specificity of Acyl-Homoserine Lactone Synthases Examined by Mass Spectrometry. J Bacteriol. 2006;188: 773–783. 10.1128/JB.188.2.773-783.2006 16385066 PMC1347284

[28] von Bodman SB, Farrand SK. Capsular polysaccharide biosynthesis and pathogenicity in Erwinia stewartii require induction by an N-acylhomoserine lactone autoinducer. J Bacteriol. 1995;177: 5000–5008. 7665477 10.1128/jb.177.17.5000-5008.1995 PMC177277

[29] Lewenza S, Visser MB, Sokol P a. Interspecies communication between Burkholderia cepacia and Pseudomonas aeruginosa. Can J Microbiol. 2002;48: 707–716. 12381027 10.1139/w02-068

[30] Hudaiberdiev S, Choudhary KS, Vera Alvarez R, Gelencsér Z, Ligeti B, Lamba D, et al Census of solo LuxR genes in prokaryotic genomes. Front Cell Infect Microbiol. 2015;5: 20 10.3389/fcimb.2015.00020 25815274 PMC4357305

[31] Passos da Silva D, Patel HK, González JF, Devescovi G, Meng X, Covaceuszach S, et al Studies on synthetic LuxR solo hybrids. Front Cell Infect Microbiol. 2015;5: 52 10.3389/fcimb.2015.00052 26151032 PMC4471428

[32] O’Loughlin CT, Miller LC, Siryaporn A, Drescher K, Semmelhack MF, Bassler BL. A quorum-sensing inhibitor blocks Pseudomonas aeruginosa virulence and biofilm formation. Proc Natl Acad Sci U S A. 2013;110: 17981–17986. 10.1073/pnas.1316981110 24143808 PMC3816427

[33] Sanchez-Contreras M, Bauer WD, Gao M, Robinson JB, Allan Downie J. Quorum-sensing regulation in rhizobia and its role in symbiotic interactions with legumes. Philos Trans R Soc Lond B Biol Sci. 2007;362: 1149–1163. 10.1098/rstb.2007.2041 17360278 PMC2435579

[34] Golberg K, Eltzov E, Shnit-Orland M, Marks RS, Kushmaro A. Characterization of quorum sensing signals in coral-associated bacteria. Microb Ecol. 2011;61: 783–792. 10.1007/s00248-011-9848-1 21523464

[35] Marketon MM, Gronquist MR, Eberhard A, González JE. Characterization of the Sinorhizobium meliloti sinR/sinI locus and the production of novel N-acyl homoserine lactones. J Bacteriol. 2002;184: 5686–5695. 10.1128/JB.184.20.5686-5695.2002 12270827 PMC139616

[36] Lindemann A, Pessi G, Schaefer AL, Mattmann ME, Christensen QH, Kessler A, et al Isovaleryl-homoserine lactone, an unusual branched-chain quorum-sensing signal from the soybean symbiont Bradyrhizobium japonicum. Proceedings of the National Academy of Sciences. 2011;108: 16765–16770. 10.1073/pnas.1114125108 PMC3189028 21949379

[37] Latifi A, Winson MK, Foglino M, Bycroft BW, Stewart GS, Lazdunski A, et al Multiple homologues of LuxR and LuxI control expression of virulence determinants and secondary metabolites through quorum sensing in Pseudomonas aeruginosa PAO1. Mol Microbiol. Wiley Online Library; 1995;17: 333–343. 7494482 10.1111/j.1365-2958.1995.mmi_17020333.x

[38] Eberhard A, Burlingame AL, Eberhard C, Kenyon GL, Nealson KH, Oppenheimer NJ. Structural identification of autoinducer of Photobacterium fischeri luciferase. Biochemistry. 1981;20: 2444–2449. 7236614 10.1021/bi00512a013

[39] Nasuno E, Kimura N, Fujita MJ, Nakatsu CH, Kamagata Y, Hanada S. Phylogenetically Novel LuxI/LuxR-Type Quorum Sensing Systems Isolated Using a Metagenomic Approach. Appl Environ Microbiol. 2012;78: 8067–8074. 10.1128/AEM.01442-12 22983963 PMC3485958

[40] Passador L, Cook JM, Gambello MJ, Rust L, Iglewski BH. Expression of Pseudomonas aeruginosa virulence genes requires cell-to-cell communication. Science. 1993;260: 1127–1130. 8493556 10.1126/science.8493556

[41] Pearson JP, Gray KM, Passador L, Tucker KD, Eberhard A, Iglewski BH, et al Structure of the autoinducer required for expression of Pseudomonas aeruginosa virulence genes. Proc Natl Acad Sci U S A. 1994;91: 197–201. 8278364 10.1073/pnas.91.1.197 PMC42913

[42] Puskas A, Greenberg EP, Kaplan S, Schaefer AL. A quorum-sensing system in the free-living photosynthetic bacterium Rhodobacter sphaeroides. J Bacteriol. 1997;179: 7530–7537. 9393720 10.1128/jb.179.23.7530-7537.1997 PMC179706

[43] Lutz R, Bujard H. Independent and tight regulation of transcriptional units in Escherichia coli via the LacR/O, the TetR/O and AraC/I1-I2 regulatory elements. Nucleic Acids Res. 1997;25: 1203–1210. 9092630 10.1093/nar/25.6.1203 PMC146584

[44] Cox RS, Madsen C, McLaughlin JA, Nguyen T, Roehner N, Bartley B, et al Synthetic Biology Open Language (SBOL) Version 2.2.0. J Integr Bioinform. 2018;0. 10.1515/jib-2018-0001 29605823 PMC6167039

[45] Canton B, Labno A, Endy D. Refinement and standardization of synthetic biological parts and devices. Nat Biotechnol. 2008;26: 787–793. 10.1038/nbt1413 18612302

[46] Ortori CA, Dubern JF, Chhabra SR, Cámara M, Hardie K, Williams P, et al Simultaneous quantitative profiling of N-acyl-l-homoserine lactone and 2-alkyl-4(1H)-quinolone families of quorum-sensing signaling molecules using LC-MS/MS. Anal Bioanal Chem. 2011;399: 839–850. 10.1007/s00216-010-4341-0 21046079

[47] Guidelines for Environmental Infection Control in Health-Care Facilities [Internet]. PsycEXTRA Dataset. 10.1037/e545922006-001

[48] Borchardt SA, Allain EJ, Michels JJ, Stearns GW, Kelly RF, McCoy WF. Reaction of acylated homoserine lactone bacterial signaling molecules with oxidized halogen antimicrobials. Appl Environ Microbiol. 2001;67: 3174–3179. 10.1128/AEM.67.7.3174-3179.2001 11425738 PMC92997

[49] Tabor JJ, Salis HM, Simpson ZB, Chevalier A a., Levskaya A, Marcotte EM, et al A Synthetic Genetic Edge Detection Program. Cell. Elsevier Ltd; 2009;137: 1272–1281. 10.1016/j.cell.2009.04.048 19563759 PMC2775486

[50] Moe-Behrens GHG, Davis R, Haynes KA. Preparing synthetic biology for the world. Front Microbiol. 2013;4: 5 10.3389/fmicb.2013.00005 23355834 PMC3554958

[51] Chen Y, Kim JK, Hirning AJ, Bennett Matthew R. Emergent genetic oscillations in a synthetic microbial consortium. Science. 2016;349: 986–989. 10.1126/science.aaa3794 PMC4597888 26315440

[52] Tashiro Y, Kimura Y, Furubayashi M, Tanaka A, Terakubo K, Saito K, et al Directed evolution of the autoinducer selectivity of Vibrio fischeri LuxR. J Gen Appl Microbiol. 2016;247: 1–8. 10.2323/jgam.2016.04.005 27725402

[53] Grant PK, Dalchau N, Brown JR, Federici F, Rudge TJ, Yordanov B, et al Orthogonal intercellular signaling for programmed spatial behavior. Mol Syst Biol. 2016;12: 849–849. 10.15252/msb.20156590 26814193 PMC4731010

[54] Knight T. Idempotent Vector Design for Standard Assembly of Biobricks [Internet]. 2003. 10.21236/ada457791

