# Supplementary material for: Characterization of diverse homoserine lactone synthases in Escherichia coli
Source: PLoS One. 2018 Aug 23;13(8):e0202294. doi: 10.1371/journal.pone.0202294 (PMC6107141; doi:10.1371/journal.pone.0202294)
Supplement: S1 Appendix — The following list includes sequences of the open reading frames for the ten synthase proteins used in this study. The FASTA header includes the symbol of the synthase (e.g. RpaI), followed by an abbreviation for the species of origin (e.g. R_palustris, R. palustris), the NCBI Accession number (e.g. WP_011155888), and length in bp (e.g. 657bp). Start (ATG) and stop codons are shown in bold font. (DOCX) [file pone.0202294.s001.docx]

**S1 Appendix. Modular Sender DNA sequences in FASTA format.**

The following list includes sequences of the open reading frames for the ten synthase proteins used in this study. The FASTA header includes the symbol of the synthase (e.g. RpaI), followed by an abbreviation for the species of origin (e.g. R_palustris, *R. palustris*), the NCBI Accession number (e.g. WP_011155888), and length in bp (e.g. 657bp). Start (ATG) and stop codons are shown in bold font.

>RpaI_R_palustris_WP_011155888_657bp
**ATG**CAGGTGCATGTGATTCGCCGTGAAAACCGCGCGCTGTACGCCGGCCTGTTAGAAAAATACTTTCGCATCCGCCATCAAATTTACGTCGTGGAGCGCGGTTGGAAGGAACTCGACCGTCCAGACGGACGCGAAATTGATCAATTCGATACCGAAGATGCAGTGTACCTGCTGGGTGTTGACAACGATGACATCGTAGCTGGTATGCGTATGGTGCCAACCACCAGCCCAACTCTCCTTAGCGATGTCTTTCCCCAACTGGCGCTGGCGGGGCCGGTGCGCCGTCCGGATGCTTATGAATTGAGTCGTATTTTTGTGGTTCCACGCAAACGTGGTGAGCATGGAGGCCCGCGCGCAGAAGCTGTGATTCAGGCTGCCGCGATGGAATACGGCCTGTCTATTGGCTTGAGTGCCTTTACCATTGTTTTAGAAACGTGGTGGCTGCCCCGTCTGGTGGATCAAGGCTGGAAGGCCAAACCGTTAGGCCTTCCACAGGATATTAATGGTTTTTCGACCACCGCCGTGATTGTGGATGTCGATGATGATGCCTGGGTCGGCATCTGCAACCGCCGTAGTGTGCCGGGGCCGACCTTGGAATGGCGCGGTTTGGAAGCAATCCGCCGCCATAGCCTTCCAGAATTTCAGGTCATCTCT**TAA**

>BraI_Bradyrhizobium_ORS278*_*WP_011924108_684bp
**ATG**CCGGAAATTCATGTAGTGCGTAAAGATAACCGCGCCCTGTATGAAAAATATTTTGATCCCTACTACCGTTTACGCCACGAGATCTATGTGAAACAGCGCAAATGGATGGATTTAGACCGCCCGGACGGGCGCGAGATTGATCAGTTCGACACTGAAGATGCGGTCTATCTGTTTTGCATTGATAACGGTCAACTGATTGGTTCAATGCGCGCAGTCCCGACTGTACTGCCGACCTTGATGAGCGATATTTTTCCTTACCTGAATCTGCGTGGCCCGGTTCAGCGCCCTGATGTCTATGAGCTCTCTCGCATTTTCGTAATCCCAGAACGTCGCGGTGAGCATGCTGGCCCGCGTATTGATATGCTGCTGCTGACTGCCATTATGGAATACGGGATTAGCATCGGCCTGACCGGTTTCTCAATCGTCCTTGAAAGCTGGTGGCTCCCCCGTTTCGAAAAATGCGGCTGGAAAGCACGTCCGCTGGGTGTACCCCATATTATGGATGGGATGTCGGTGCTGGCTGTTCTGGTCGATTGCGACGAAACCACATGGAAATCACTGTGCACCCAGATCGGTCTGACCCGTCCTACCTTAACTTGGCAGGGATTAGAGGAAGTGTCTCGCCAAGCTCTCCCTGATATTTTCTTGCACCTGCCACCGGCCGTGCAACCCGCACAG**TAA**

>RhlI_Pseudomonas_WP_003113896_609bp
**ATG**ATCGAACTGCTGTCCGAATCCCTGGAAGGTCTGTCCGCTGCTATGATCGCTGAACTGGGTCGTTACCGTCACCAGGTTTTCATCGAAAAACTGGGTTGGGACGTTGTTTCCACCTCCCGTGTTCGTGACCAGGAGTTCGACCAGTTCGACCACCCGCAGACCCGTTACATCGTTGCTATGTCCCGTCAGGGTATCTGCGGTTGCGCTCGTCTGCTGCCGACCACCGACGCTTACCTGCTGAAAGACGTTTTCGCTTACCTGTGCTCCGAAACCCCGCCGTCCGACCCGTCCGTTTGGGAACTGTCCCGTTACGCTGCTTCCGCTGCTGACGACCCGCAGCTGGCTATGAAAATCTTCTGGTCCTCCCTCCAGTGCGCTTGGTACCTGGGTGCTTCCTCCGTTGTTGCTGTTACCACCACCGCTATGGAACGTTACTTCGTTCGTAACGGTGTTATCCTCCAGCGTCTGGGTCCGCCGCAGAAAGTTAAAGGTGAAACCCTGGTTGCTATCTCCTTCCCGGCTTACCAGGAACGTGGTCTGGAAATGCTGCTGCGTTACCACCCGGAATGGCTCCAGGGTGTTCCGCTGTCCATGGCTGTTTAA**TAA**

>BjaI_Bradyrhizobium_WP_011083883_672bp
**ATG**GGCGTATCGATGATTCATGCAATTAGCGCCGTGAACCGCCATCTTTATGAGGATGTACTGGAGCAGCATTTTCGTCTTCGCCATGATATTTTCGTAGAGGAGCGTCACTGGGAAACGCTGCGCCGCCCAGATGGGCGCGAGGTTGATAGTTACGATGATGAAGATACGGTATATCTTTTAGCTTTAGAAGGCCGCCGTGTCGTAGGGGGGCATCGTCTGTATCCGACCACAAAACCGTCCATGATGAGCGAGGTGTTTCCCCATCTCGCTGCAGTGCGTGGCTGTCCCTCGGATCCCCTGATTTGGGAATGGTCACGTTATTTCGTTGTGCGTGACCGTCGTGATGGTGCGCTGAATCTGCAGCTGATGGCCGCGGTACAGGAGTTTTGCCTCGATCAGGGGATTGCCCAAGTGTCCGCCATTATGGAAACATGGTGGCTTCCGCGTTTTCACGAAGCTGGGTTTGTGGTCACACCGCTGGGGTTACCAGCCCTGGTCGAGAATGCCTGGACTATGGCTGCAACAGTTGACATCCGTCGTCAGACCCTGGACGTCCTGCACGATCGTATTGGTATGCCGTCGATCGTCCAACAAGATGGCCCGCGTCTGGATGCAGTAGCGCGTGCAAATCTGTGCGGGCTGGCTGCAGCTCAGCGTAAGTCTGCT**TAA**

>EsaI_P_stewartii_WP_006119202_633bp
ATGCTGGAACTGTTTGATGTGTCCTATGAAGAACTGCAAACGACCCGTAGCGAAGAACTGTATAAGCTGCGTAAGAAGACCTTTAGCGATCGTCTGGGCTGGGAAGTGATTTGCAGTCAGGGTATGGAATCCGATGAATTTGACGGTCCGGGCACCCGCTATATTCTGGGCATCTGCGAGGGTCAGCTGGTTTGTTCAGTCCGTTTCACCTCGCTGGATCGCCCGAACATGATTACCCATACGTTTCAACACTGTTTCTCCGACGTCACCCTGCCGGCCTATGGCACGGAAAGCAGCCGTTTCTTTGTGGACAAAGCGCGTGCCCGCGCACTGCTGGGTGAACATTACCCGATCAGCCAGGTGCTGTTTCTGGCTATGGTTAACTGGGCTCAAAACAATGCGTATGGCAATATTTACACCATCGTTAGCCGTGCGATGCTGAAGATTCTGACGCGCTCTGGTTGGCAGATTAAAGTCATCAAGGAAGCCTTCCTGACCGAAAAAGAACGTATCTACCTGCTGACGCTGCCGGCAGGCCAAGATGACAAGCAGCAACTGGGCGGTGATGTGGTTAGTCGCACCGGTTGCCCGCCGGTCGCAGTGACCACGTGGCCGCTGACGCTGCCGGTTTAA

>LuxI_V_fischeri_CAA68562_585bp
**ATG**ACTATAATGATAAAAAAATCGGATTTTTTGGCAATTCCATCGGAGGAGTATAAAGGTATTCTAAGTCTTCGTTATCAAGTGTTTAAGCAAAGACTTGAGTGGGACTTAGTTGTAGAAAATAACCTTGAATCAGATGAGTATGATAACTCAAATGCAGAATATATTTATGCTTGTGATGATACTGAAAATGTAAGTGGATGCTGGCGTTTATTACCTACAACAGGTGATTATATGCTGAAAAGTGTTTTTCCTGAATTGCTTGGTCAACAGAGTGCTCCCAAAGATCCTAATATAGTCGAATTAAGTCGTTTTGCTGTAGGTAAAAATAGCTCAAAGATAAATAACTCTGCTAGTGAAATTACAATGAAACTATTTGAAGCTATATATAAACACGCTGTTAGTCAAGGTATTACAGAATATGTAACAGTAACATCAACAGCAATAGAGCGATTTTTAAAGCGTATTAAAGTTCCTTGTCATCGTATTGGAGACAAAGAAATTCATGTATTAGGTGATACTAAATCGGTTGTATTGTCTATGCCTATTAATGAACAGTTTAAAAAAGCAGTCTTAAATTAA**TAA**

>SinI_Sinorhizobium_WP_003534106_645bp
**ATG**ATCCGTATTGTAAACGGCAACGGTCGTTCTCAGCACCCTCAGGCCATTGACGAAATGTTTCGCTTACGTAAACGCGTGTTTCATGATTTTCTTAAATGGGATGTTAAAACCGAAGGGGATTGGGAAATTGACCATTACGATAAAGCTAATCCGTTATACGTAATGAGCTATAGTCCAGATACGGGTAAAATCCGTGGTTCTTTGCGTCTGCTGCCCACACTGGGACCGAACATGCTGGATGATACCTTCCCGATTCTGCTGGGTGACAATCCGGAAATTCGCTCTGCTAGCGTCTGGGAATCGAGCCGCTTCTGTATCGATCCAGAAATTAGTCAGGATCGCGCGTCGAACCAAGTAACGATTGCGGCTGCGGAATTAATGTGCGGTGTGGGTGAAATGTCGCTGGCAAGCGGTATCTCGCATATTGTGACCGTCACGGATGTGTTCCTGGAGCGTATGTTCCGTCGCATGGGTTGTCCGGGCGAACGCATTGCAGACCCACACCGCATTGGTTCGGTTCATGCGGTCGCGATTGCGTGGGAAGTGAGTCGCAATCTGCTTGAAACTATGAAGGCTGTCGCCTCCATTGAAGGCACGGTTCTGGATCGTCCCATGTCTTTGGAAACTGCGCGCGCGGCC**TAA**

>AubI_unknown_BAM45368_714bp
**ATG**CGTCGCGTGAAAGACGTGGAGTTCTCCGAAAACGGCTTTGTAGTCAAGACCCTGCATGGCGGTCAATTAGCCCAGTCATACCGTCTGCGTCATAAAGTCTTCGCGGAAAGCTTGAAATGGGTCCCGGAGACTGAGGATCGCCAAGAAATCGACCTGTACGATCTGTGGGCGACCACGGTGGGTTTAGTGCGCGACGACGGGGCTGTGGTGGGTGTGGCGCGTCTGCTTCCAAGCAGTAGTCAGTTCATGCTGGAAAAAGAATTTGGCGCACTTTTGCCTTGCGGATATCAGATCCGTAAAGGTCCGGATACAGCGGAGATCACACGCTTGGCAGTGGACCCTGATATTCGTGATCGCGGGCTGAGTTCCCGCATGATGCTGGCGCTGTTGAAAGGTGTTTATCAGTGGGCGGTAGAAAACGACATCCGTTATTACTACCTGGAGGTGGAACATCGCTTTTTTCGTGCACTGCGTGCCTTGGGCTTTCCATGTGAAATGATTGGCGAACCGGTGGTCCTGCCGCCAGCAGGCGCTAGCAGCGTTGCAGCACTGTATGATATGGTCCGCTTTGATGAAGAAAACGCCATTAAGAAACCACAGTTCCTGAAGTGGATCAGCAGTATCGAGACTCTGCAAGGCGAGGTGATTGCGGGTCGTACGTCCTCCTACGCGAACTCCGAAAAATTAGGCCTGGTGGGCGCCGAGGCC**TAA**

>LasI_Pseudomonas_WP_003083017_609bp
**ATG**ATCGTTCAGATCGGTCGTCGTGAAGAGTTCGACAAAAAACTGCTGGGTGAAATGCACAAACTGCGTGCTCAGGTTTTCAAAGAACGTAAAGGTTGGGACGTTTCCGTTATCGACGAAATGGAAATCGACGGTTACGACGCTCTGTCCCCGTACTACATGCTGATCCAGGAAGACACCCCGGAAGCTCAGGTTTTCGGTTGCTGGCGTATCTTCGACACCACCGGTCCGTACATGCTGAAAAACACCTTCCCGGAACTGCTGCACGGTAAAGAAGCTCCGTGCTCCCCGCACATCTGGGAACTGTCCCGTTTCGCTATCAACTCCGGTCAGAAAGGTTCCCTGGGTTTCTCCGACTGCACCCTGGAAGCTATGCGTGCTCTGGCTCGTTACTCCTTGCAGAACGACATCCAGACCCTGGTTACCGTTACCACCGTTGGTGTTGAAAAAATGATGATCCGTGCTGGTCTGGACGTTTCCCGTTTCGGTCCGCACCTGAAAATCGGTATCGAACGTGCTGTTGCTCTGCGTATCGAACTGAACGCTAAAACCCAGATCGCTCTGTACGGTGGTGTTCTGGTTGAACAGCGTCTGGCTGTTTCCTAA**TAA**

>CerI_R_sphaeroides_WP_011338011_634bp
**ATG**ATCTTCATTATTGACAGCCTTAACTTGCGCGAGCACGCTGACATCGTCAAAGACATGTTCCGCTTGCGCAAGCGTGTCTTCGCTGATCGCCTGGGTTGGGATGTACAAATCTCTCAAGGAATGGAACGCGACCGCTTCGATGACTTGGACCCCGCGCATGTTGTGAGTGTGGATGATGAAGGTCGTGTCGTAGGGTGTATGCGCCTGTTGCAAACCACCGGCCCACATATGCTGTCCGACGTGTTTTCGAGTATCCTGGATGGTGAACCGCCGCTGCGTAGCGCCACATTATGGGAAGCGACCCGCTTCTGCGTTGATACTGATCGTCTGGTGTCTGGACGCGCACGTAATAGCATCGCATACGTGACCTCGGAAGTTATGATTGGCGCTTTCGAATTCGCGATGTCCGCGGGCGTGACGGACGCTGTTGCGGTGATTGACCCGGTCATGGACCGTGTGCTGAAACGCTCAGGCAATGCCCCGCAGGGATATGTTGGTACTCCGAAACCCATGGGTAAGGTCACTGCTCTGGCGGCTTTGATGGATTGCTCCGAAGAACGCGTCAAGCGCATTCGCGATTTTGCAGGCATCTATCACGATGTTACCCAACCGCAGACGGTAATCGCT**TAA**C
