# Supplementary material for: Characterization of diverse homoserine lactone synthases in Escherichia coli
Source: PLoS One. 2018 Aug 23;13(8):e0202294. doi: 10.1371/journal.pone.0202294 (PMC6107141; doi:10.1371/journal.pone.0202294)
Supplement: S1 Table — “Antiquity” indicates that no specific team is known to have contributed the DNA sequence to the Registry. Entries can be accessed at http://parts.igem.org/ (DOCX) [file pone.0202294.s002.docx]

| **Part name** | **iGEM Registry part number** | **Contributing iGEM Team** |
| --- | --- | --- |
| RpaI | BBa_K1421006 | iGEM14_CAU_China |
| BraI | BBa_K2033004 | iGEM16_Arizona_State |
| RhlI | BBa_C0170 | Antiquity |
| BjaI | BBa_K2033002 | iGEM16_Arizona_State |
| EsaI | BBa_K1670004 | iGEM15_Manchester-Graz |
| LuxI | BBa_C0161 | Antiquity |
| SinI | BBa_K2033008 | iGEM16_Arizona_State |
| AubI | BBa_K2033000 | iGEM16_Arizona_State |
| LasI | BBa_C0078 | Antiquity |
| CerI | BBa_K2033006 | iGEM16_Arizona_State |
| LuxR receiver | BBa_F2620 | MIT |

**S1 Table.** **Relevant iGEM Registry IDs.** “Antiquity” indicates that no specific team is known to have contributed the DNA sequence to the Registry. Entries can be accessed at <http://parts.igem.org/>
