# Supplementary material for: Characterization of diverse homoserine lactone synthases in Escherichia coli
Source: PLoS One. 2018 Aug 23;13(8):e0202294. doi: 10.1371/journal.pone.0202294 (PMC6107141; doi:10.1371/journal.pone.0202294)
Supplement: S1 Fig — Sender cells were grown overnight in liquid media, pelleted, and lysed before electrophoresis of whole protein lysate for size analysis. Sender cell lysates were electrophoresed on a 4–12% Bis-Tris polyacrylamide gel at 200 V for 50 minutes in 1x MOPS buffer. Gels were stained for 1 hour with coomassie dye R-250 (Imperial Protein Stain, Thermo fisher #24615) for one hour, followed by two hours of destaining in water. The first lane of each gel is a standard ladder (Invitrogen #LC5800); 30, 20, and 15 kDa bands are marked. Expected protein sizes were calculated using CLC Main Workbench ver. 8.0.1 Protein Report tool. Red arrows indicate the mCherry protein (expected size 26.7 kDa). White arrows indicate expected synthase protein bands. (A) A comparison of lysates from Control-EGFP cells and LuxI Sender (pTetR-LuxI-mCherry) cells shows bands that correspond with the expected sizes for mCherry (26.7 kDa) and LuxI (22.3 kDa) only in the LuxI Sender lysate. The bottom image shows band intensity traces generated by the ImageJ (ver. 1.51s, Macintosh) Gel Analyzer tool. (B) Lysates from the other nine Senders were subjected to PAGE in two trials (top gel, BjaI and LasI 2 μL per lane, others 5 μL; bottom gel, 2 μL lysate per lane). (C) Band intensity traces for the gels shown in B. Data from the lanes in gel 2 (bottom) were arranged to match the order of synthases for gel 1 (top). The trace from a non-transformed control (blue) is overlaid with pTetR-mCherry (red) to distinguish the mCherry peak. For other lanes, the pTetR-mCherry trace is overlaid with Sender (pTetR-Synthase-mCherry) traces (grey). (DOCX) [file pone.0202294.s003.docx]

**
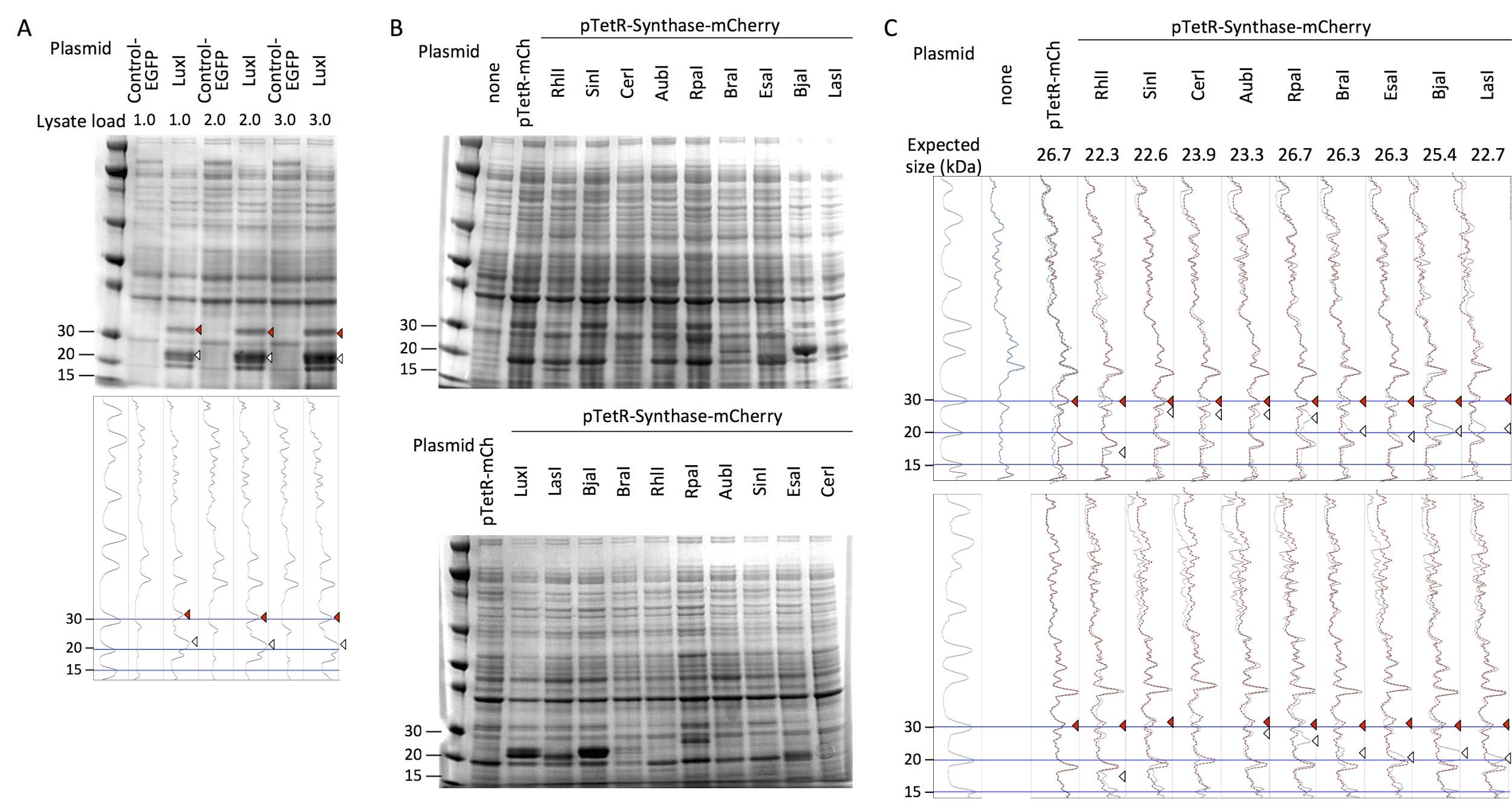
**

**S1 Figure. PAGE analysis of lysates from Sender transformants.** Sender cells were grown overnight in liquid media, pelleted, and lysed before electrophoresis of whole protein lysate for size analysis. Sender cell lysates were electrophoresed on a 4-12% Bis-Tris polyacrylamide gel at 200 V for 50 minutes in 1x MOPS buffer. Gels were stained for 1 hour with coomassie dye R-250 (Imperial Protein Stain, Thermo fisher #24615) for one hour, followed by two hours of destaining in water. The first lane of each gel is a standard ladder (Invitrogen #LC5800); 30, 20, and 15 kDa bands are marked. Expected protein sizes were calculated using CLC Main Workbench ver. 8.0.1 Protein Report tool. Red arrows indicate the mCherry protein (expected size 26.7 kDa). White arrows indicate expected synthase protein bands. (A) A comparison of lysates from Control-EGFP cells and LuxI Sender (pTetR-LuxI-mCherry) cells shows bands that correspond with the expected sizes for mCherry (26.7 kDa) and LuxI (22.3 kDa) only in the LuxI Sender lysate. The bottom image shows band intensity traces generated by the ImageJ (ver. 1.51s, Macintosh) Gel Analyzer tool. (B) Lysates from the other nine Senders were subjected to PAGE in two trials (top gel, BjaI and LasI 2 μL per lane, others 5 μL; bottom gel, 2 μL lysate per lane). (C) Band intensity traces for the gels shown in B. Data from the lanes in gel 2 (bottom) were arranged to match the order of synthases for gel 1 (top). The trace from a non-transformed control (blue) is overlaid with pTetR-mCherry (red) to distinguish the mCherry peak. For other lanes, the pTetR-mCherry trace is overlaid with Sender (pTetR-Synthase-mCherry) traces (grey). Red arrows indicate the mCherry protein (expected size 26.7 kDa). White arrows indicate presumed synthase protein bands (strongest non-mCherry band).

**Method: Preparation of Sender lysates for PAGE analysis.** 3.0 mL of Luria Broth (LB) supplemented with 100 μg /mL ampicillin was inoculated with a single Sender colony and grown at 37℃, 220 RPM overnight (16 hours). Cultures were diluted to OD_600_ = 2.0 and 1.0 mL of cells was pelleted and resuspended in 75 μL H_2_0 and 25 μL 4x loading dye (Invitrogen NP0007). Resuspended cells were heated at 95℃ for 5 minutes and spun through a 0.22 μm cellulose acetate filter (Costar 8161) before being loaded onto a gel. 10 μL protein ladder (Invitrogen #LC5800 ) was used as a standard.
