# Supplementary material for: Characterization of diverse homoserine lactone synthases in Escherichia coli
Source: PLoS One. 2018 Aug 23;13(8):e0202294. doi: 10.1371/journal.pone.0202294 (PMC6107141; doi:10.1371/journal.pone.0202294)
Supplement: S2 Fig — The plots show the unnormalized data from Fig 4: Control-GFP cells grown in mock-enriched medium (from pTetR-mCh cells); left, Absorbance (OD600) over time; right, unnormalized GFP signal over time. EGFP and optical density (OD600) were measured every 10 minutes for 240 minutes (4 hours). Graphs show means of triplicate wells (bars, standard deviation). We observed that the relative GFP values (signal divided by OD600) decreased over time (Fig 4B). However, the raw data show that total GFP expression increases over time during culture growth. (DOCX) [file pone.0202294.s004.docx]

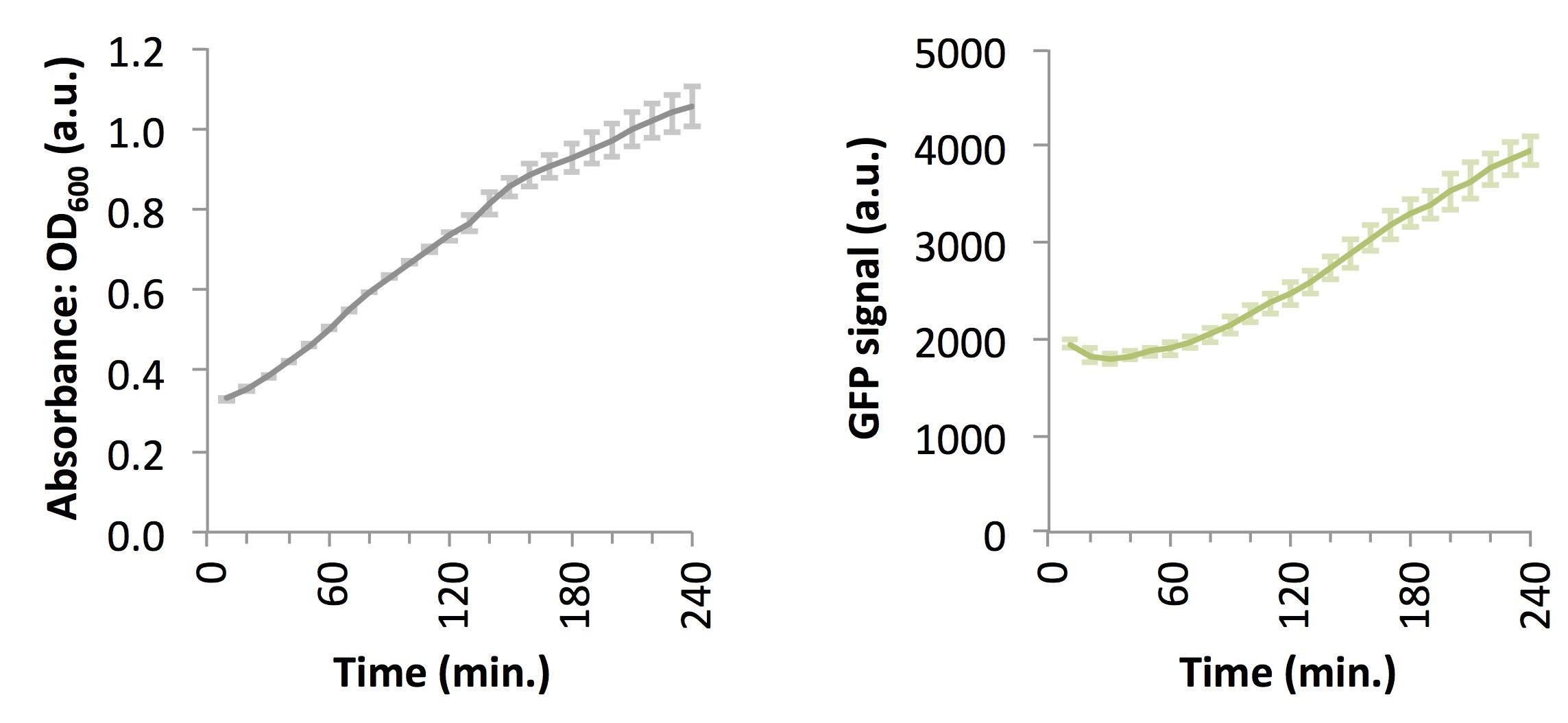


**S2 Fig. EGFP expression from Control-EGFP over time.**  The plots show the unnormalized data from Figure 3: Control-EGFP cells grown in mock-enriched medium (from pTetR-mCh cells); left, absorbance (OD_600_) over time; right, unnormalized GFP signal over time. EGFP and optical density (OD_600_) were measured every 10 minutes for 240 minutes (4 hours). Graphs show means of triplicate wells (bars, standard deviation). We observed that the relative GFP values (signal divided by OD_600_) decreased over time (Fig 3B). However, the raw data show that total GFP expression increases over time during culture growth.
